# The challenge of unidentified decedents in Africa: The need for training and research in forensic odontology to strengthen a multidisciplinary approach

**DOI:** 10.3389/froh.2022.1017736

**Published:** 2022-09-26

**Authors:** Susan Chandler, Stephen M. Sudi, Keané C. Bailie, Manogari Chetty

**Affiliations:** ^1^ Faculty of Dentistry, University of the Western Cape, Bellville, Cape Town, South Africa; ^2^ University of the Western Cape/ University of Cape Town Combined Dental Genetics Clinic, Red Cross Children’s Hospital, Cape Town, South Africa

**Keywords:** forensic odontology, oral health, sub-Saharan africa, dental records, unidentified decendents

## Abstract

**Introduction:**

The management of unidentified decedents suspected to be undocumented migrants is a growing humanitarian crisis in Africa. Identification of the dead and the right of the family to know the fate of a decedent is a fundamental human right. Forensic odontology methods can provide helpful and assisting information in the identification even in challenging low-resource settings. South Africa and other countries that are part of significant migration routes face the problem of unidentified decedents.

**Discussion:**

The fundamental application of forensic odontology relies on the availability of good antemortem dental records. The state of dental records was reported to be suboptimal in South Africa and other African countries. Incorporating forensic odontology into the undergraduate training in the 23 dental schools in Africa will increase the understanding of the value of maintaining accurate dental records and potentially facilitate collaboration with dentists and forensic odontologists in cases where dental features can be used for identification. South Africa offers postgraduate training in forensic odontology, and prospects for research in Africa need to be explored.

**Conclusion:**

The development of a forensic odontology career path and research prospects will provide African countries with the potential for building multidisciplinary teams to assist in solving the challenge of unidentified decedents.

## Introduction

The management of unidentified decedents suspected to be undocumented migrants is a growing humanitarian crisis facing forensic pathology services in African countries (1–4). The driving forces for migration in Africa, namely disasters, conflict, repressive governance, and limited economic opportunities (5), predispose migrants to several risks and even death (6–8). The disappearance of a migrant is known to cause an impact at an individual, family, and community level. A missing migrant's relatives are left plagued by psychosocial issues such as the need to discover a loved one's fate, find closure, and social recognition of the grief (9). Intervention modalities for the psychosocial issues related to the disappearance are often unavailable due to hostile forces which drive migration in Africa (10). An estimated 1.1 billion individuals worldwide cannot prove their identity, with more than 40 per cent of those lacking means of identification residing in Africa (11). The lack of identification documents in those forcibly displaced may lead to using illegal means to migrate, further making them susceptible to abuse, exploitation, and difficulties in identification in cases of demise.

Sub-Saharan Africa (SSA), the poorest and least developed Sustainable Development Goal Region (12) is vulnerable to natural and human-induced hazards and disasters that severely impact lives and livelihoods (13). Sub-Saharan Africa had 27.2 million internally displaced people in 2021, 46% of the global total. Of those, 25.1 million were displaced due to conflict and violence, and a further two million due to disasters (14). In 2020, 21 million Africans were living in other countries, with South Africa being the most desirable destination in Africa, hosting around 2.9 million international migrants (14).

In recognition of the magnitude of violations of the fundamental rights of migrants and refugees, the African Union established the Migration Policy Framework and Plan of Action(2018–2030) (15), and the African Commission on Human and People's Rights adopted a resolution 486 on missing migrants in Africa and the impact on their families (16). The Geneva convention articles provide provisions for the identification of the dead, and the right of the family to know the fate of a decedent in conflict situations as a fundamental human right (17, 18).

The current reports on missing migrants in Africa originate from non-governmental organisations' databases. The reports reveal marked variation in the numbers of missing migrants; nonetheless, all point to a growing humanitarian crisis. To illustrate, the International Organization for Migration stated that 1,697 migrants had been reported missing since 2014 (19). The International Committee of the Red Cross database indicated that 44,000 migrants were missing from 2010 to 2020, (10) and the Associated Press revealed that 18,400 migrants have been missing or dead since 2014 (20).

South Africa is one of eight upper-middle-income countries in Africa and an attractive destination for both internal (rural to urban) and international migrants (21). South Africa's forensic pathology services in major metropolitan areas is burdened by unidentified decedents. The migrants who are unsuccessful in seeking opportunities to better their lives and assimilate into society may end up homeless (22). Crime and violence are rampant in South Africa's metropolitan areas (23). The combination of high crime levels, high unemployment rates, and lack of documentation contributes to the difficulties in identifying deceased individuals (2, 24). The unknown and unidentified individuals may be buried by the state and appear among the missing databases. This cannot be confirmed because statistics and other data regarding unidentified bodies and missing people are not formally gathered (25).

The recent media commentaries allude to the high number of unknown and unclaimed bodies from Gauteng – 898 bodies (26), Cape town – 472 bodies (27), and Durban - over 200 bodies (28). The International Organization for Migration reported 685 deaths and disappeared during migration in Southern Africa between 2014 and 2021 (29). In a retrospective review, Reid et al. (2020) described a high percentage of unidentified individuals (9.2%) at Salt River Mortuary, which serves the Western Metropole of the City of Cape Town. The review recommended facilitated communication between parties and scientific researchers to ensure social justice and lessen the burden of unidentified persons.

The International Committee of the Red Cross established a project to assist the South African Police Services and Forensic Pathology Services with identifying the unknown decedents in the Johannesburg Medicolegal Laboratory, Gauteng province, in 2015. The multidisciplinary project utilised forensic photography, radiology, dentistry, dactylography, DNA analysis, and forensic anthropology (30). By 2021, the project conducted 416 examinations of unidentified bodies, with 93 confirmed identities, 49 were South Africans and 44 foreigners (31). This project's multidisciplinary and collaborative approach can serve as a model for Africa in general. The success of the multidisciplinary approach calls for increased training, research, and collaboration in forensic odontology in Africa. This review aims to describe the potential of forensic odontology' in contributing towards alleviating the humanitarian crisis of unidentified decedents and documenting the various niche areas within the discipline that require further research.

## Forensic odontology

Forensic odontology is “The branch of dentistry that deals with the legal aspects of professional dental practices and treatment, with particular emphasis on using dental records to identify victims of crimes or accidents” (32). It is concerned with identifying human beings employing dentition and craniofacial features.

Forensic odontology has played a significant role in natural mass disasters such as earthquakes, tsunamis, floods, tornados, hurricanes, and wildfires (33, 34) and man-made disasters of wars, conflicts, acts of terrorism, and transportation accidents (33). Forensic odontology can also be utilised in assaults, murder, human abuse, and neglect cases (33, 35, 36).

Forensic odontology has a rich history, with the first notable report of Agrippina the Younger, a case from AD 49. In the 15th century, Charles the Bold, the Duke of Burgundy from 1467 to 1477, was killed in war. He was identified by his Italian page primarily by some missing teeth (33). In 1776, Dr Joseph Warren, who was killed in the Battle of Bunker Hill, was identified by his dentist, Paul Revere, through a small denture fixed with a silver thread (33, 37). The inclusion of dentists as expert witnesses occurred in 1814 (33). While in 1848, the field of forensic odontology was first used as evidence in court in the case of Parkman-Webster (37). There are multiple cases in which forensic odontology has been notably used in horrific scenarios, such as the historic fire of Bazar de la Charite, Paris, in 1897, where the remains of the victims were charred beyond recognition, and dental comparisons were used to determine identities (33).

Forensic odontology officially started in South Africa in 1966, following the appointment of the first Honorary Forensic Odontologist Police Consultant (38). In identifying victims, forensic odontologists have contributed in various instances, such as the 1968 Windhoek aviation tragedy, which marked the beginning of the practical application of forensic odontology in South Africa (39). Apart from identification, the field expanded in 1983 after Professor H.A. Shapiro founded the first specialised forensic dental publication in South Africa (38). The Forensic odontologists who have contributed to Forensic odontology in South Africa over the years include Professor C.W. Van Wyk, Professor A.J. Ligthelm, Professor V.M. Phillips and Professor H. Bernitz (38). This rich history highlights the use and importance of forensic odontology.

In more recent times, forensic odontology expertise has been utilised in limited instances in mass disaster cases occurring in Africa. In the crash of Dana Air Flight 0992 in Nigeria, only 10% of the victims were identified through forensic odontology due to poor or total lack of dental records (34). Since then, a dentist from Lagos has trained to become the first forensic odontologist in West Africa (40).

Scientific publications reporting on forensic odontology in Africa primarily emanate from Southern Africa. These include studies on age estimation of children (41–43), adult dental age estimation (44), sex determination (45, 46), and dental records (47–50). Historical factors related to the need to identify victims of frequent acts of violence contributed to the advancement of forensic odontology in South Africa (51).

### Facets of forensic odontology

The modern discipline of forensic odontology is comprised of several facets. The first and most common facet involves the morphological and visual aspects, which include dental identification, sex determination, age estimation, bite-mark analysis, lip print analysis, palatal rugae analysis and metric and nonmetric morphological traits of dentition (45, 46, 52–54). Secondly, dental photography utilises both postmortem and antemortem images. The images include photos showing the victim's face, and especially the teeth (55, 56). The third aspect is radiography which includes ante and postmortem radiographs of the ‘victim's dentition and craniofacial features (52). The fourth aspect is advanced biomolecular methods which may be used for age estimation, the establishment of provenance, and gender determination. The advanced biomolecular methods include aspartic acid racemisation, carbon dating, advanced glycation end products, the composition of teeth, collagen crosslinks, lead accumulation, mitochondrial deletions, DNA methylation, signal joint t-cell receptor excision circles in human lymphoid tissues (sjTRCs), telomere shortening, isotope, and DNA analysis (57–63).

## Discussion

### Challenges with regards to dental records

The fundamental application of forensic odontology relies on the availability of good antemortem dental records. These records strongly rely on good oral health services and legislative mechanisms which enforce the proper recording of patient data and management details.

Unfortunately, oral health services in Africa are characterised as a low priority due to the prevalence of numerous general health issues and significant developmental needs (64).

Oral health services are inadequate, resulting in predominantly extractions to relieve discomfort or pain as the primary treatment. An increase in tooth loss among adult individuals has been documented (65), and this directly impacts the use of dental comparison for identification. Dental comparison identification can utilise one unique-shaped restoration to identify a victim positively (66).

The state of dental records in South Africa was reported to be suboptimal, and the situation is unlikely to be different in other African countries. A study at MEDUNSA Oral Health Centre, a dental training institution in South Africa, described the standard of dental records and record-keeping to be below the Health Professions Council of South Africa (HPCSA) requirements. In this study, 25 per cent of the selected records did not contain the details of the clinical procedures performed on the patients (47). In a study from Cape Town, records from dentists in private practice were close to optimal, while those from lower socio-economic areas were inadequate (50). South Africa does not have legal frameworks guiding the proper recording and maintenance of dental records. The introduction of legal frameworks will enforce improved record-keeping standards. In a report from Uganda, dental students' perception of record keeping was described as low, and the study recommended incorporating the importance of record keeping into the teaching and learning aspect of the curriculum (48).

The poor state of record keeping has an impact, whether unintentional or intentional, in cases of insurance fraud, impacts negatively on the utility of dental records for identification purposes and may lead to the employment of costly identification methods such as DNA analysis, further increasing the cost of the process and delaying closure for the relatives of the deceased (67).

Incorporating forensic odontology into the undergraduate training in the 23 dental schools in Africa (64), will increase understanding of the value of maintaining accurate dental records and potentially facilitate collaboration with dentists and forensic odontologists in cases where dental features can be used for identification.

The status and state of oral health in the world and Africa are likely to improve with the global drive toward universal health coverage by 2030 (68) and the release of the WHO Oral Health Strategy (69). The milestone achievements will improve oral health training, services, and research. The achievements provide an opportunity to concomitantly improve forensic odontology services, given that when adequate records are available, dental identification is cost-effective, as evident in Thailand during the boxing day tsunami of 2006 (70).

In developing countries, despite the lack of adequate dental records, forensic odontologists can still provide expertise in identifying victims of disasters. Skinner *et al*. (2010) observed inaccuracies in dental examinations where forensic odontologists were not involved and described situations where forensic odontologists' observations were corroborated by surviving relatives in the absence of dental records, resulting in a successful identification (71).

### Postgraduate training

In South Africa, postgraduate courses in forensic odontology are offered at the University of the Western Cape (72) and the University of Pretoria (73). The courses are at the level of postgraduate diploma (PGDip), Master's (MSc) degree, and PhD degree. At the University of the Western Cape, the postgraduate diploma aims at enabling the qualified dentist to assist in the identification of unknown victims and act as an expert witness. It also provides sufficient knowledge and practical skills to assist the professional in mass disasters, bite mark cases, and human abuse and neglect. The MSc degree supplements the PGDip syllabus by focusing on developing research skills in the field, while the PhD is geared towards academics and developing research niches relevant to the region. The research areas for the MSc and PhD students focus on building the local knowledge base and examples of studies from South Africa and Sudan (46, 74). The information on the different courses is available on the universities' websites (72, 73).

### Potential research areas

The African continent has niches with a potential for further research. The deliberate and ritualistic mutilation of the dentition has been reported in several ethnic groups of Africa. Mutilation patterns include avulsions/extractions, shape modifications, and decorations (75). The current practices in the mutilation of dentition need to be researched and documented as such attributes can be used to identify ethnicity. The great rift valley bisects the African continent, the geology of which has rendered water sources with high fluoride levels. High fluoride levels in portable water similarly occur in isolated areas on the African continent, leading to an increased prevalence and severity of dental fluorosis (76). Dental fluorosis can be used to determine provenance during infancy. The distribution of isotopes with utility in provenance in Africa is not known. Irish J (2013) described Afridonty as a group of nonmetric morphological traits of dentition as differentiation features in sub-Saharan Africans (52). Research on these nonmetric morphological features of dentition and their utility in forensic odontology is lacking, and this could be an area where forensic odontology and anthropology collaboration will provide identification traits for different subgroups of sub-Saharan Africans.

## Conclusion and recommendations

Africa is a continent of developing countries with a multiethnic population and cultures. It is also a land of much discord resulting in large numbers of displaced people, many missing and frequently unidentified. Thus, there is a critical requirement for African governments to establish networks and population-specific databases, which may aid as a point of entry in the identification of individuals.

Establishing collaborations and partnerships among African forensic odontologists may be a way forward in providing solutions due to the cross-border and international nature of the migrant crisis.

Although Africa bears up to 90% of the global disease burden, it only has access to about 10% of global health research funding. African governmental financing of dentists who wish to pursue postgraduate degrees in forensic odontology is mainly unavailable, which now warrants urgent focus. This would encourage the development of expertise within the field of forensic odontology at various African dental schools.

It is also recommended that dental training at the core undergraduate curriculum should include the basics of forensic odontology embedded within, which is the need for good dental record maintenance. This would enhance interest in the field of forensic odontology as well as improve the competency of the dentist. In addition, postgraduate interest in the field may also be inspired by an improvement in research capacity necessary in Africa for Africans. The development and use of population and ethnic group-specific forensic odontology databases can assist in providing provenance details during victim identification.

Forensic odontology forms an integral part of forensic sciences and is a cost-effective body of knowledge for identifying human remains through dentition and craniofacial features. The forensic odontologist is a highly trained specialist capable of providing valuable inputs to the medico-legal team comprised of forensic pathologists, forensic anthropologists, district surgeons, attorneys and police officers. Efforts to enhance the awareness of the police force, forensic pathology services and other disciplines (*e.g.* Forensic Anthropology) in the identification of decedents using Forensic odontology should be initiated, and this will strengthen a multidisciplinary approach.

## Data Availability

The original contributions presented in the study are included in the article/Supplementary Material, further inquiries can be directed to the corresponding author/s.
